# Current causes of death in familial hypercholesterolemia

**DOI:** 10.1186/s12944-022-01671-5

**Published:** 2022-08-02

**Authors:** Victoria Marco-Benedí, Ana M. Bea, Ana Cenarro, Estíbaliz Jarauta, Martín Laclaustra, Fernando Civeira

**Affiliations:** 1 Hospital Universitario Miguel Servet, IIS Aragón, CIBERCV, Zaragoza, Spain; 2 Universidad de Zaragoza, Zaragoza, Spain; 3 Unidad de Lípidos, Hospital Universitario Miguel Servet, Avda Isabel La Católica 1-3, Zaragoza, 50009 Spain; 4 Instituto Aragonés de Ciencias de La Salud (IACS), Zaragoza, Spain

**Keywords:** Heterozygous familial hypercholesterolemia phenotype, Parental-offspring, Cardiovascular disease death

## Abstract

**Background:**

Familial hypercholesterolemia (FH) is a codominant autosomal disease characterized by high low-density lipoprotein cholesterol (LDLc) and a high risk of premature cardiovascular disease (CVD). The molecular bases have been well defined, and effective lipid lowering is possible. This analysis aimed to study the current major causes of death of genetically defined heterozygous familial hypercholesterolemia (heFH).

**Methods:**

A case‒control study was designed to analyse life-long mortality in a group of heFH and control families. Data from first-degree family members of cases and controls (nonconsanguineous cohabitants), including deceased relatives, were collected from a questionnaire and review of medical records. Mortality was compared among heFH patients, nonheFH patients, and nonconsanguineous family members.

**Results:**

A total of 813 family members were analysed, 26.4% of whom were deceased. Among the deceased, the mean age of death was 69.3 years in heFH individuals, 73.5 years in nonheFH individuals, and 73.2 years in nonconsanguineous individuals, without significant differences. CVD was the cause of death in 59.7% of heFH individuals, 37.7% of nonheFH individuals, and 37.4% of nonconsanguineous individuals (*P* = 0.012). These differences were greater after restricting the analyses to parents. The hazard ratio of dying from CVD was 2.85 times higher (95% CI, (1.73–4.69) in heFH individuals than in individuals in the other two groups (non-FH and nonconsanguineous), who did not differ in their risk.

**Conclusions:**

CVD mortality in heFH individuals is lower and occurs later than that described in the last century but is still higher than that in non-FH individuals. This improved prognosis of CVD risk is not associated with changes in non-CVD mortality.

**Supplementary Information:**

The online version contains supplementary material available at 10.1186/s12944-022-01671-5.

## Introduction

Familial hypercholesterolemia (FH) is an autosomal codominant disorder and the most common monogenic metabolic disease. The prevalence of heterozygous FH (heFH) is approximately 1/200–500 persons in most countries [1, 2]. FH is caused by mutations in the genes that control the cellular uptake of plasma cholesterol and that include the LDL receptor (*LDLR),* apolipoprotein B *(APOB),* pro-protein convertase subtilisin/kexin 9 *(PCSK9)* and *APOE* [1, 3, 4]. HeFH patients show a very high plasma concentration of low-density lipoprotein cholesterol (LDLc), approximately twice that of non-FH subjects in the general population and often ranging between 250 and 400 mg/dL. They also often show deposits of cholesterol in superficial tissues, such as corneal arcus and tendon xanthomas, and a high risk of premature cardiovascular disease (CVD) in the absence of adequate lipid-lowering treatment [5, 6]. The risk of developing premature CVD is increased by a factor of 10 in these patients with respect to the general population, especially in the case of coronary heart disease (CHD) in young patients [6]. International heFH registries, such as the British Simon Broome, show an up to 100 times higher risk of CHD in men under 40 years of age with heFH in the prestatin era; a treatment that was not available until the late 1980s [7]. Additionally, the life expectancy of individuals with heFH has been calculated to be between 10 and 30 years lower for women and men than for the non-FH population [8]. In recent years, there has been a decrease in CVD in heFH patients, and it has been recently verified in our environment [6, 9], probably due to earlier diagnosis and intensive lipid-lowering treatment.

Two important findings have been identified in the morbidity and mortality analysis of heFH in recent decades. First, the genetic bases of heFH have been studied in depth, and genetic study provides a certain diagnosis that removes the diagnostic bias based on CVD risk as one of the major criteria for heFH diagnosis [10]. Second, current lipid-lowering drugs, including statins, ezetimibe and PCSK9 inhibitors, have substantially modified the lipid phenotype and consequently the clinical spectrum of the disease [11, 12]. In this way, if the treatment is well established during the first decades of life, heFH should be a less aggressive disease than before. The complexity of the genetic FH background, the use of multiple drugs for decades, a longer life expectancy associated with treatment and changes in environmental and social factors could lead to a much more heterogeneous phenotype than that described in the past century [1, 3]. In addition, other comorbidities, such as diabetes, could be associated with the heFH phenotype that is masked by CVD or associated with lipid-lowering treatment, often including statins [13]. Knowing the effects of the different genetic types of heFH in the long term and the impact of prolonged lipid-lowering treatment is essential for adequate management of this disease in the coming years.

The aim of this analysis was to study the current causes of cardiovascular and noncardiovascular deaths in heFH subjects and the potential differences when compared to a control population.

## Patients and methods

### Aim, design, and participants

This was an observational, case‒control study designed to describe the current morbidity and mortality situation in heFH subjects. HeFH cases were recruited from the Lipid Unit at Hospital Universitario Miguel Servet, Zaragoza, Spain, and their nonconsanguineous partners were recruited as controls. Data about first-degree family members of cases and controls, including deceased relatives, were collected using a participant questionnaire from 2019 to 2021 and a review of medical records from 2001 onwards. Written informed consent was obtained from each case and control included in the study; the study protocol conforms to the ethical guidelines of the 1975 Declaration of Helsinki and was previously approved by the Institution's ethics committee on research on humans (Comité Ético de Investigación Clínica de Aragón).

The inclusion criteria for cases consisted of the following requirements: age ≥ 30 and ≤ 60 years at the time of enrolment in the study, genetically diagnosed heFH, and personal history of hypercholesterolemia with LDLc levels > 220 mg/dL without lipid-lowering treatment. Controls were selected from nonconsanguineous relatives of heFH patients of a similar age (± 5 years) (partners of the case who cohabited with them); the control inclusion criteria were as follows: age ≥ 30 and ≤ 60 years at the time of inclusion in the study, no first-degree relative or personal clinical diagnosis of heHF and LDLc < 190 mg/dL without lipid-lowering drugs.

#### Clinical interview

Participants were interviewed to collect personal information about the history of CVD disease, CV risk factors, comorbidities, medication use, lipid values, and hospitalizations and to obtain a detailed family history including the same data for all first-degree relatives (parents, siblings, and offspring) and the age and cause of death of those deceased. Information on hypercholesterolemia history, lipid-lowering drug use, age, and cause of death was confirmed from the patient’s medical records. If a first-degree family member presented LDLc > 220 mg/dL on at least one occasion and/or LDLc > 160 mg/dL while taking any statin, they were considered to belong to the heFH group. The analysis groups were thus “heFH family members”, “nonheFH family members”, and “control family members”. In this report, only data on family members deaths that occurred over the age of 18 years are presented. CVD was defined as coronary (myocardial infarction, coronary revascularization procedure, sudden death); cerebral (stroke); peripheral vascular disease (intermittent claudication, amputation or arterial revascularization); or abdominal aortic aneurysm. CVD death included any death that was the result of any CVD event as we defined.

#### Genetic study

All heFH cases interviewed in this study had a genetic diagnosis of heFH and were carriers of a "pathogenic" or "likely pathogenic" variant according to the guidelines of the American College of Medical Genetics and Genomics (ACMG) [14] in the *LDLR* (NM_000527.4), *APOB* (NM_000384.2) or *PCSK9* (NM_174936.3) genes. All heFH cases underwent sequencing of exon 4 of *APOE* (NM_000041.4), as previously described [4]. FH gene analyses were studied with the Progenika Biopharma Grifols (Derio, Spain) [15] or GEN inCode (Terrassa-Barcelona, Spain) [16] platforms.

### Statistical analyses

Data are expressed as the mean standard deviation for numerical variables with a normal distribution, which were analysed with Student's T test, while those without a normal distribution are expressed as the median [interquartile range] and were analysed with the Mann–Whitney U test. Qualitative variables are expressed as a percentage and were analysed using the X^2^ test. For the comparison of nondichotomous categorical variables, ANOVA and Kruskal–Wallis tests were used. The mortality rates were calculated using the Kaplan–Meier estimate based on age, and the groups were compared using the log rank test. The associations between heFH and CV and non-CVD mortality were calculated using multivariate Cox regression. A model was generated that included the covariate age, and it was calculated with techniques appropriate for analysing complex samples considering that data were clustered in families.

## Results

### Clinical characteristics of cases and controls

The study recruited 166 subjects: 83 heFH cases and 83 controls. The genes responsible for heFH were *LDLR* in 78 subjects, *APOB* in 4 subjects and *APOE* in 1 subject. The mean ages were 54.3 years and 54.5 years, respectively, without differences in age and sex between the groups. BMI, systolic blood pressure and diastolic blood pressure were similar in both groups. Hypertension and diabetes (DM2) prevalence showed no differences either. CVD tended to be more prevalent in heFH cases than in controls (8.4% and 2.4%, respectively) (*P* = 0.08). Untreated total cholesterol and LDLc were higher in cases than in controls. Statin treatment was present in all cases and in 22.9% of controls. The onset of lipid-lowering treatment was 32.8 years in heFH patients and 51.3 years in controls (Table 1).

**Table 1:** Clinical and biochemical characteristics of heFH and control probands

	**heFH Cases**	**Controls**	***P***
N	83	83	
Age (years)	54.3 (10.7)	54.5 (10.5)	0.884
Women, N (%)	45 (54.2)	44 (53.0)	0.876
Current smokers, N (%)	11 (13.3)	22 (26.5)	0.098
BMI (Kg/m^2^)^a^	25.8 (3.94)	26.3 (4.17)	0.479
Systolic blood pressure (mmHg)	123.1 (12.7)	123.3 (14.0)	0.956
Diastolic blood pressure (mmHg)	74.6 (8.97)	75.3 (9.97)	0.628
Hypertension, N (%)	24 (28.9)	16 (19.8)	0.172
Type 2 diabetes mellitus, N (%)	7 (8.4)	7 (8.4)	1.000
Cardiovascular disease, N (%)	7 (8.4)	2 (2.4)	0.087
Total cholesterol (mg/dL)	363 (412–306)	220 (198–252)	< 0.001
LDL cholesterol (mg/dL)^b^	283 (222–339)	130 (105–154)	< 0.001
HDL cholesterol (mg/dL)^c^	56.2 (13.4)	62.0 (24.2)	0.079
Triglycerides (mg/dL)	114 (52.3)	141 (154)	0.209
Glucose (mg/dL)	89.5 (18.8)	91.3 (20.1)	0.626
Statin treatment, N (%)	83 (100)	19 (22.9)	< 0.001
Onset age of statin treatment (years)	32.8 (9.43)	51.3 (8.84)	< 0.001

Data are summarized as mean (SD) or N (percentage)

^a^BMI denotes body mass index

^b^LDL, low-density lipoprotein

^c^HDL, high-density lipoprotein

### Family study

**Fig. 1:**
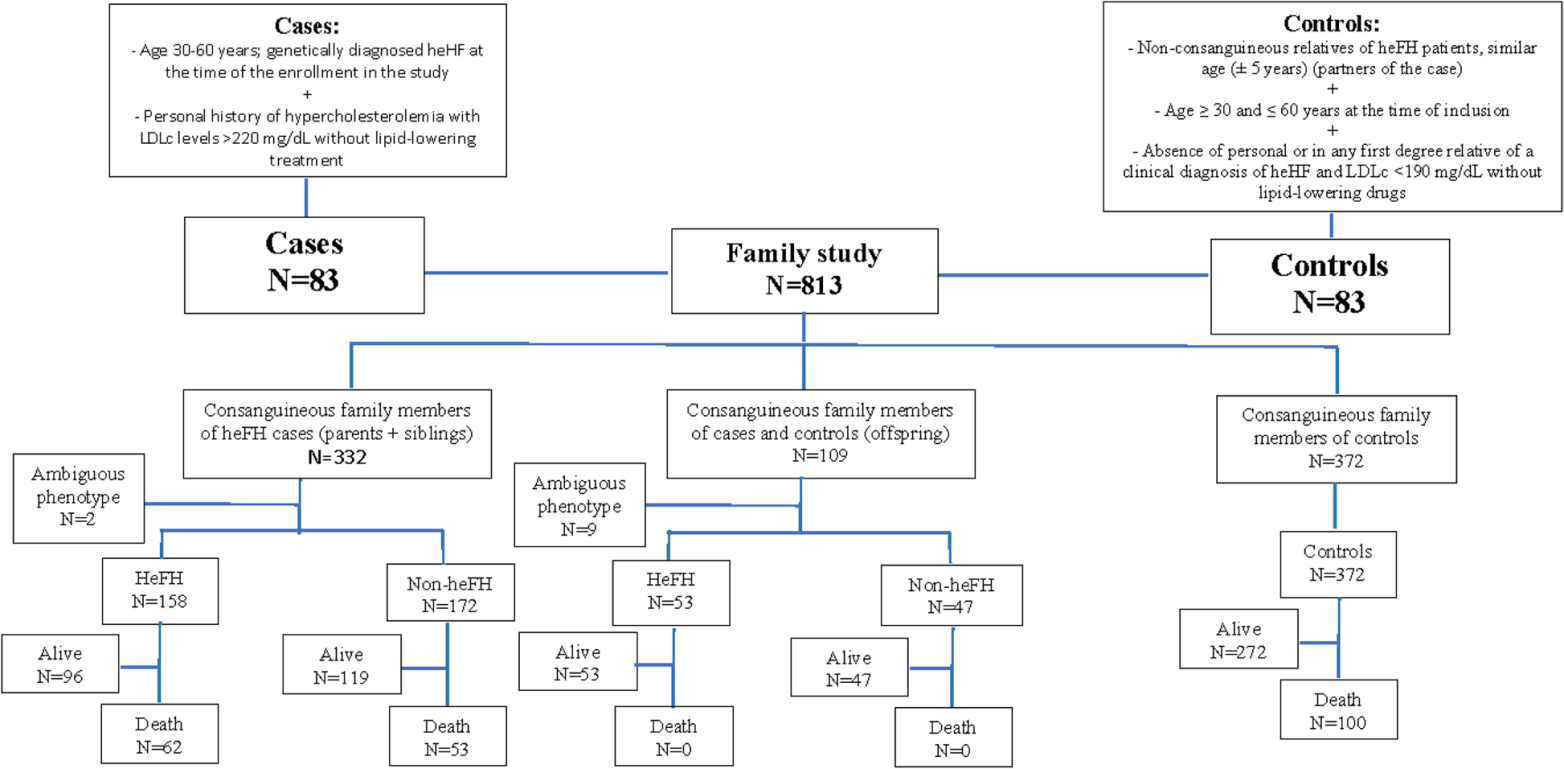
Study Flowchart

A total of 813 first-degree family members of cases and controls were analysed, and it was found that within families of cases, 211 members had heFH and 219 did not have heFH (Supplemental Table 1). Eleven first-degree family members of cases with an ambiguous heFH phenotype were excluded (Fig. 1). The control family group was composed of 372 subjects.

### CVD and non-CVD mortality among first-degree family members of cases and controls

**Fig. 2:**
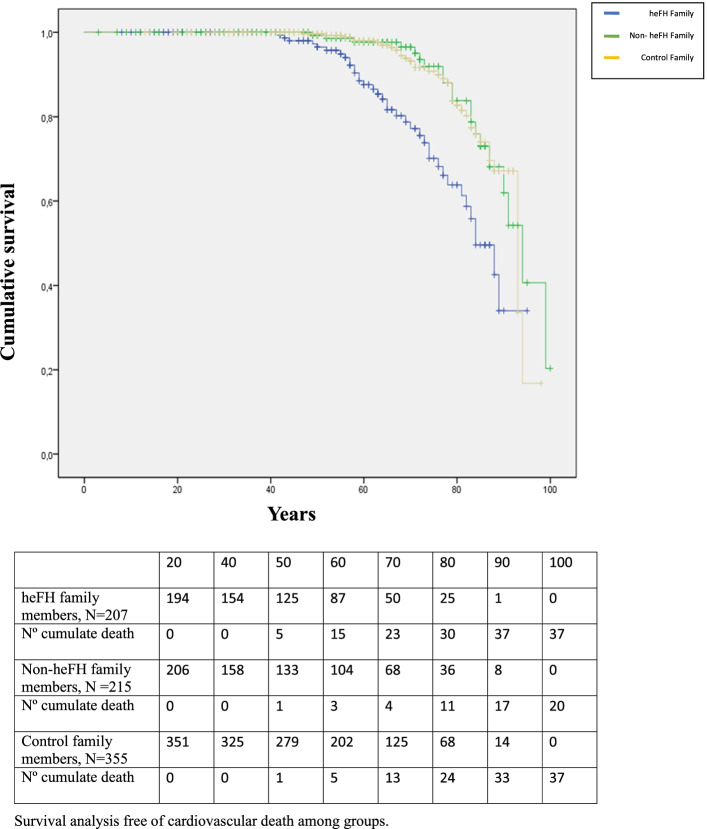
Kaplan–Meier cumulative survival curves for cardiovascular death

The family study identified 62 dead relatives among heFH family members: 53 among nonheFH individuals and 100 among controls (Fig. 1). The percentage of dead subjects and the mean age of death were similar in the three groups, being slightly higher in the heFH family members, 29.4% compared with 24.2% in the nonheFH family members and 26.9% in the control family members. The average age of death was approximately 4 years younger in the heFH group, and the mean age of CVD death was 65.9 years in the heFH group, 77.5 years in the nonheFH group and 77.5 years in the control group. The proportion of deaths due to CVD was higher in the heFH group (59.7% in heFH vs. 37.7% in nonheFH and 37.4% in controls, *P* = 0.012). Cerebrovascular death was included within CVD death and occurred in 7 (11.3%) heFH subjects, 5 (9.4%) nonheFH individuals and 10 (10.0%) controls, without differences among groups. Other causes of death, including cancer, did not show significant differences among the three groups (Table 2). The most frequent cancer responsible for deaths was that of digestive origin (35.4%), followed by breast and prostate cancers (29.2%) and lung cancer (22.9%), with no differences between groups, although the number of cancer cases by subgroups was limited. Additionally, mortality differences between men and women were analysed. The percentage of deceased subjects did not show differences between the groups; however, HeFH subjects died approximately 4 years earlier than nonheFH individuals and controls, although the difference was not statistically significant. The cause of death in men was CVD in 69% of heFH individuals versus 38.5% of nonheFH individuals and 37.0% of controls (P = 0.01). The same pattern was observed in women, although the age of death was approximately 7 years older in women than in men in the 3 groups. (Table 3 and Table 4). The hazard ratio of dying of CVD was 2.85 times higher (95% CI, 1.73–4.69) in the heFH group than in the control family group, and there were no differences between the nonFH and control groups. This hazard ratio was 2.95 in men (95% CI, 1.52–5.75) and 3.44 in women in the heFH group (95% CI, 1.66–7.10) (Table 5). The separation of the curves appeared at the age of 50 and continued increasing progressively with age (Fig. 2). This higher risk appeared approximately 5 years earlier in heFH men than in heFH women (Supplemental Fig. 1).

**Table 2:** Mortality among heFH and control first-degree family members

	**heFH family members**	**NonheFH family members**	**Control family members**	***P***
N	211	219	372	
Total death, N (%)	62 (29.4)	53 (24.2)	100 (26.9)	0.479
Age of death (years)	69.3 (13.9)	73.5 (16.2)	73.2 (13.8)	0.179
Age of Cardiovascular disease death (years)	65.9 (13)	77.5 (12)	77.5 (13.2)	0.001
Age of Non-cardiovascular death (years)	73.9 (13.8)	71.4 (17.6)	71.72 (14.6)	0.792
Cardiovascular disease death, N (%)	37 (59.7)	20 (37.7)	37 (37.4)	0.012
Non-cardiovascular death, N (%)	25 (40.3)	33 (62.3)	62 (62.6)	
Cancer death, N (%)	12 (48.0)	14 (42.0)	31 (50.0)	0.779
Other death, N (%)	13 (52.0)	19 (57.5)	31 (50.0)	
Statin treatment, N (%)	107 (50.7)	29 (13.2)	53 (14,2)	0.001

Data are summarized as mean (SD) or N (percentage). Test for raw differences using Chi^2^ test

**Table 3:** Mortality among heFH and control first-degree male family members

	**heFH family members**	**NonheFH family members**	**Control family members**	***P***
N	100	115	187	
Total death, N (%)	29 (29.0)	39 (34.2)	65 (34.8)	0.599
Age of death (years)	65.8 (13.0)	72.2 (16.1)	70.7 (14.4)	0.099
Age of Cardiovascular disease death (years)	62.2 (11.5)	78.7 (13)	71.2 (11.6)	0.001
Age of Non-cardiovascular death (years)	73.7 (13)	68.0 (17)	70.4 (15.8)	0.650
Cardiovascular disease death, N (%)	20 (69.0)	15 (38.5)	24 (37.0)	0.010
Non- cardiovascular death, N (%)	9 (31)	24 (61.5)	41 (63.1)	
Cancer death, N (%)	6 (66.7)	12 (50)	19 (46.3)	0.543
Other death, N (%)	3 (33.3)	12 (50.0)	22 (53.6)	

Data are summarized as mean (SD) or N (percentage). Test for raw differences using Chi^2^ test

**Table 4:** Mortality among heFH and control first-degree female family members

	**heFH family members**	**NonheFH family members**	**Control family members**	***P***
N	111	104	185	
Total death, N (%)	33 (29.7)	14 (13.5)	34 (18.4)	0.010
Age of death (years)	72.4 (14.1)	77.2 (16.6)	78.1 (11.5)	0.178
Age of Cardiovascular disease death (years)	70.2 (13.5)	74 (15.0)	82.6 (8.7)	0.032
Age of Non-cardiovascular death (years)	74.1 (14.6)	80.3 (17.6)	74.3 (11.7)	0.513
Cardiovascular disease, N (%)	17 (51.5)	5 (35.7)	13 (38.2)	0.451
Non- cardiovascular death, N (%)	16 (48.5)	9 (64.3)	21 (61.8)	
Cancer death, N (%)	6 (37.5)	2 (22.2)	12 (57.1)	0.175
Other death, N (%)	10 (62.5)	7 (77.7)	9 (42.8)	

Data are summarized as mean (SD) or N (percentage). Test for raw differences using Chi^2^ test

**Table 5:** Prospective multivariable Cox Regression Analysis of Predictive Factors for a cardiovascular death in the families group

**heFH family members**	**CVD death HR (95% CI)**	**CVD death HR (95% CI)**^**a**^
All	3.02 (1.90–4.79)	2.85 (1.73–4.69)
Males	2.90 (1.59–5.29)	2.95 (1.52- 5.71)
Females	3.20 (1.55–6.63)	3.44 (1.66–7.10)
**NonheFH family members**	**HR (95%CI)**	**HR (95%CI)**^**a**^
All	0.81 (0.46–1.42)	0.98 (0.58–1.65)
Males	0.79 (0.49–1.57)	0.80 (0.41–1.53)
Females	0.95 (0.33–2.67)	1.02 (0.38–2.71)

95% CI, 95% confidence interval; HR, hazard ratio. HR (95%CI)

^a^confidence interval estimations calculate taking into account family clusters

### CV or non-CVD mortality among parental family members of cases and controls

**Fig. 3:**
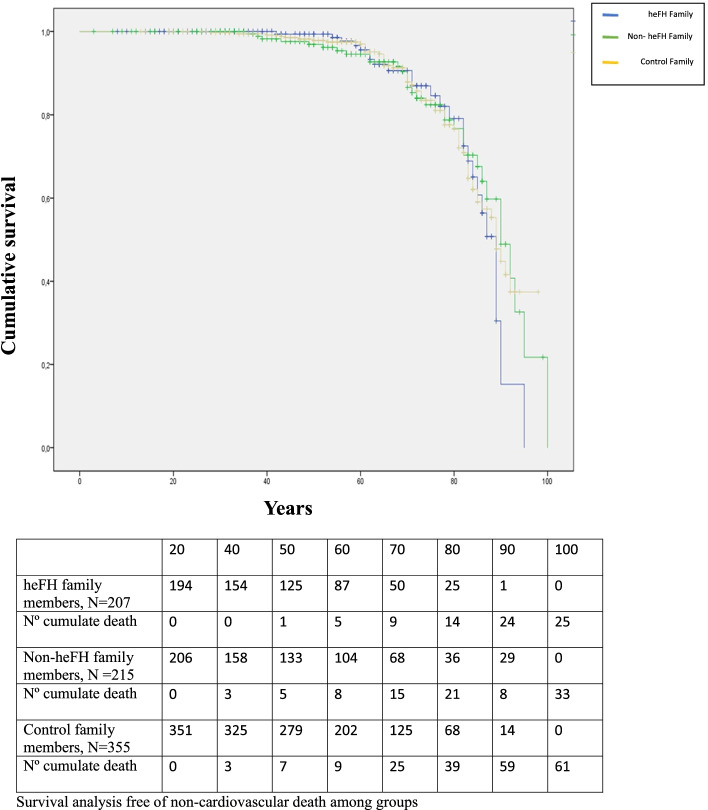
Kaplan–Meier cumulative survival curves for noncardiovascular death

Since most of the deaths corresponded to the parents of cases and controls, they were analysed separately. There were 116 deaths among fathers: 24 (72.7%) in heFH individuals, 35 (72.9%) in nonheFH individuals and 57 (70.4%) in controls. A total of 77 deaths occurred among mothers: 33 (66.0%) in heFH individuals, 13 (39.4%) in nonheFH individuals and 31 (37.8%) in controls. The percentage of deaths from CVD was higher in the heFH group than in the other two groups, although the difference was significant only in men, and the age of death from CVD was younger in both men and women for heFH subjects. There were no statistically significant differences in non-CVD death (Fig. 3), but the control mothers had a trend towards higher cancer death compared to that in heFH mothers (*P* = 0.092) (Supplemental Tables 2 and 3).

## Discussion

In this research, the potential differences in mortality in a group of heFH families from a lipid unit, comparing heFH patients, nonheFH patients, and nonconsanguineous family members, were analysed with the aim of updating CVD and non-CVD deaths in the heFH group in the era of lipid-lowering treatment. HeFH is a singular model used to study the effect of hypercholesterolemia on mortality due to an increase in LDLc levels and their relationship with CVD events [17]. In addition, heFH patients are usually under intensive chronic lipid-lowering treatment, and the results are in line with the CVD benefit observed with LDLc lowering in the general population [18]. The study hypothesized that the prevalence of CVD death has decreased during the last years in these patients, and the results seem to support this idea because CVD mortality in this group of families is lower and appeared later than that in the heFH cohorts in the last decades of the last century [3, 19]. However, CVD death is still increased with respect to nonheFH, especially in the heFH men, who died 6.8 years younger than individuals in the other family groups. Traditionally, it has been considered that in heFH patients without lipid-lowering treatment, approximately 50% of men and 30% of women will develop CVD before 50 years of age [3, 19], with the life expectancy estimated to be 20 to 30 years lower [8]. However, CVD death could have been biased in those studies. Historically, heFH has been diagnosed clinically based on LDLc elevation, premature personal and familial CVD, and the presence of tendon xanthomas or arcus cornealis. The most common criteria for diagnosis, including those of the Dutch Lipid Clinic Network [20] and Simon Broome registry [21], include risk factors for CVD, such as tendon xanthomas [3, 22]. In this way, the heFH subjects or their families in whom CHD prevailed had more chances to obtain the clinical diagnosis of FH. The genetic characterization of FH in recent years has demonstrated that the heFH phenotype is more heterogeneous than previously believed, including the presence of CVD. In a recent publication from The Netherlands, CHD was present in 7.4% of 25,137 genetically diagnosed heFH patients, despite the mean age being 38 years, and 71.1% were not on lipid-lowering drugs [23]. Consequently, a significant proportion of heFH may have gone unnoticed while applying traditional diagnostic criteria.

The cohort studied is based on very high LDLc levels (> 220 mg/dl without lipid-lowering therapy) and a positive genetic test for a causative mutation in a canonical gene for FH. Furthermore, patients were referred to the clinic by their general practitioners because of high LDLc. Therefore, this cohort has overcome previous bias [24]. Robust evidence, including from heFH observational studies, has demonstrated a reduction in major CVD events in patients who are taking lipid-lowering treatment when initiated early in life and maintained for years [11, 25]. Accordingly, survival without CHD, with an early initiation of statin treatment in these subjects, could be quite similar to the rest of the population [26]. This study included a large group of heFH patients who were taking lipid-lowering treatment, on average, for more than 21.5 years. Furthermore, the majority of their heFH family members had been taking statins at some point in their lives. In addition, the prevalence of CVD estimated in this study, 7% in heFH patients, is in line with other studies in genetically defined heFH [23, 27]. The study was not designed to evaluate the mechanism of the CVD reduction in heFH in recent years. Earlier diagnosis and treatment have probably contributed to this CVD reduction. In accordance with this hypothesis, a recent report by the Spanish Atherosclerosis Society Registry clearly showed that the early onset and the number of years under statin treatment were two major independent risk factors associated with a better prognosis of CVD in heFH patients [9]. The same explanation was previously suggested by the investigators of the Simon Broome Registry in the UK [21].

This study also analysed non-CVD mortality in these genetically defined heFH families with a large history of lipid-lowering drug consumption for two purposes: first, to check whether lipid-lowering therapy could play a role in other comorbidities, and second, to explore whether the FH-causing mutation itself might be associated with morbidities other than CVD once heFH subjects live long enough without CVD, something that, until now, would have been hidden by early mortality. In this study, non-CVD mortality did not show significant differences between the heFH and non-FH groups in either sex. There was a tendency in the heFH females to die later from non-CVD causes compared to non-FH patients, even though the difference did not reach statistical significance. We hypothesized that this could be due to healthier lifestyles in heFH subjects, as has been previously shown [28].

### Strengths and limitations

The strengths of this article include that all heFH cases had been genetically confirmed and that HeFH diagnosis was independent of CVD; all first-degree relatives were included and their mortality was reviewed through their medical records.

On the other hand, this study has some limitations. Its retrospective design means that only heFH patients who lived long enough were selected, so heFH subjects who died before the analysis could not be studied. The number of subjects studied, imposed by the difficulty of finding a large series of patients, allows us to identify differences in mortality in the large disease groups; however, if some rare disease is associated with the mutation or the treatment, this could have gone unnoticed. Finally, the study included information about the time of treatment onset, but only in cases and controls could it be completely corroborated.

## Conclusion

In conclusion, these results show that current CVD mortality in heFH patients is lower and occurs later than that described in the last century but is still higher than that in non-FH patients. This is probably due to better control of the risk of CVD risk factors, especially prolonged lipid-lowering treatment. This better prognosis in regard to CVD risk is not associated with changes in non-CVD mortality.

These results suggest that the clinical management of subjects with heFH should emphasize the importance of both early diagnosis and another onset of early lipid-lowering treatment in the heFH population to avoid the excess risk of CVD that is still present.

## Supplementary Information

**Additional file 1: Supplemental Figure 1.** Panel A) Kaplan–Meiercumulative survival curves for cardiovascular death in heFH male familymembers. B) Kaplan–Meier cumulative survival curves for cardiovascular death inheFH women family members.

**Additionalfile 2: Supplemental Table 1.** Clinical and biochemicalcharacteristics of heFH, non-FH and control family members. **SupplementalTable 2.**Mortality among heFH and control fathers. **SupplementalTable 3.** Mortality among heFH and control mothers.

## Data Availability

Data available upon reasoned request.

## Abbreviations

BMI: Body mass index
CHD: Coronary heart disease
CVD: Cardiovascular disease
DM: Diabetes mellitus
FH: Familial hypercholesterolemia
heFH: Heterozygous familial hypercholesterolemia
HDLc: High-density lipoprotein cholesterol
LDLc: Low-density lipoprotein cholesterol
*LDLR*: Low-density lipoprotein receptor
Lp(a): Lipoprotein(a)
PCSK9: Pro-protein convertase subtilisin/kexin 9
