# Supplementary figures and images for: Current causes of death in familial hypercholesterolemia
Source: Lipids Health Dis. 2022 Aug 2;21:64. doi: 10.1186/s12944-022-01671-5 (PMC9344778; doi:10.1186/s12944-022-01671-5)

**heFH men family members**

**A**

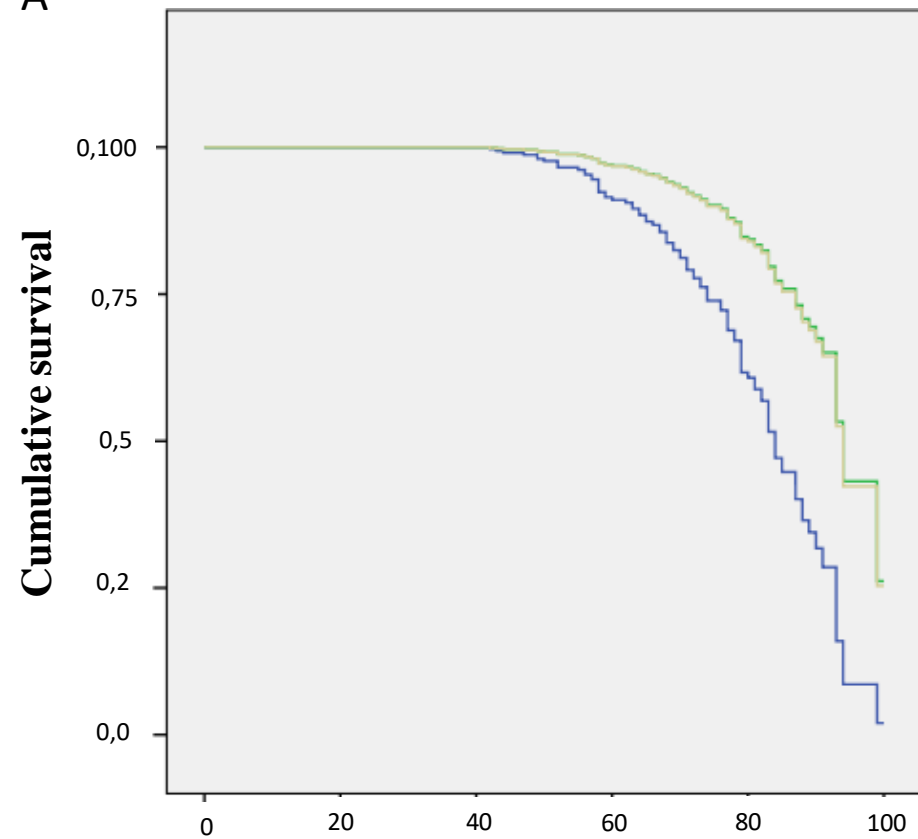

**Years**

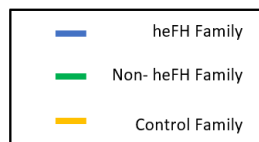

**heFH women family members**

**B**

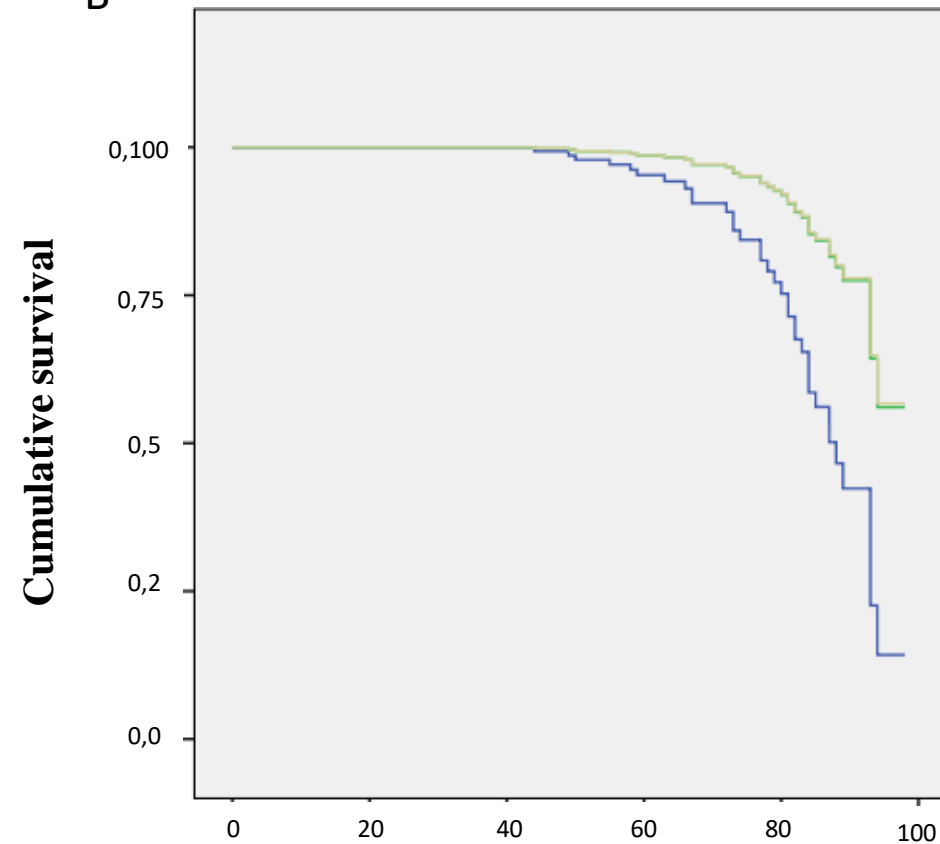

**Years**

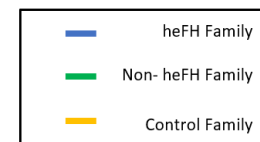

Supplement: Supplementary file 1 — Additional file 1: Supplemental Figure 1. Panel A) Kaplan–Meiercumulative survival curves for cardiovascular death in heFH male familymembers. B) Kaplan–Meier cumulative survival curves for cardiovascular death inheFH women family members. [file 12944_2022_1671_MOESM1_ESM.pdf]
