# Supplementary material for: Current causes of death in familial hypercholesterolemia
Source: Lipids Health Dis. 2022 Aug 2;21:64. doi: 10.1186/s12944-022-01671-5 (PMC9344778; doi:10.1186/s12944-022-01671-5)
Supplement: Supplementary file 2 — Additionalfile 2: Supplemental Table 1. Clinical and biochemicalcharacteristics of heFH, non-FH and control family members. SupplementalTable 2.Mortality among heFH and control fathers. SupplementalTable 3. Mortality among heFH and control mothers. [file 12944_2022_1671_MOESM2_ESM.docx]

**Supplemental Table 1. Clinical and biochemical characteristics of heFH, non-FH and control family members.**

|  | **heFH family members** | **Non-heFH family member** | **Controls**  **Family members** | ***P*** |
| --- | --- | --- | --- | --- |
| N | 211 | 219 | 371 |  |
| Age (years) | 54.6 (20.3) | 57.3 (21.6) | 63.8 (17.1) | <0.001 |
| Women, N (%) | 111 (52.6) | 104 (47.5) | 185 (49.9) | 0.568 |
| Current smokers, N (%) | 37 (17.5) | 41 (18.7) | 78 (21.0) | 0.021 |
| BMI (Kg/m^2^) | 24.5 (4.7) | 25.9 (4.9) | 27.5 (5.6) | <0.001 |
| Hypertension, N (%) | 31 (16.2) | 30 (19.5) | 95 (28.3) | 0.017 |
| Type 2 diabetes mellitus, N (%) | 23 (11.4) | 21 (10.2) | 52 (14.4) | 0.294 |
| Cardiovascular disease, N (%) | 57 (27.7) | 22 (10.6) | 53 (15) | <0.001 |
| Statin treatment, N (%) | 107 (50.7) | 29 (13.7) | 52 (14.0) | <0.001 |
| Statin treatment (years) | 11.34 (6.7) | 6.6 (4.7) | 6.7 (4.4) | <0.001 |

^a^BMI denotes body mass index; ^b^LDL, low-density lipoprotein; ^c^HDL, high-density lipoprotein; Data are summarized as mean (SD) or N (percentage). Test for raw differences using Chi^2^ test

**Supplemental Table 2. Mortality among heFH and control fathers.**

| Subject | **heFH family members** | **Non-heFH family members** | **Control family members** | ***P*** |
| --- | --- | --- | --- | --- |
| N | 33 | 48 | 81 |  |
| Total dead, N (%) | 24 (72.7) | 35 (72.9) | 57 (70.4) | 0.941 |
| Age death (years) | 68.75 (12.05) | 74.11 (15.22) | 73.70 (11.22) | 0.217 |
| Age of Cardiovascular disease death (years) | 64.5 (10.7) | 76.7 (13.0) | 71.3 (11.8) | 0.006 |
| Age of Non-cardiovascular death (years) | 79.0 (8.7) | 71 (15.7) | 75.3 (10.6) | 0.373 |
| Cardiovascular disease, N (%) | 17 (70.8) | 16 (40.4) | 23 (40.4) | 0.040 |
| Non-cardiovascular death, N (%) | 7 (29.2) | 19 (54.3) | 34 (59.6) |  |
| Cancer death, N (%) | 4 (57.1) | 8 (42.1) | 17 (50.0) | 0.759 |
| Other death, N (%) | 3 (42.8) | 11 (57.9) | 17 (50.0) |  |

Data are summarized as mean (SD) or N (percentage). Test for raw differences using Chi^2^ test

**Supplemental Table 3. Mortality among heFH and control mothers.**

| Subject | **heFH family members** | **Non-heFH family members** | **Control family members** | ***P*** |
| --- | --- | --- | --- | --- |
| N |  |  |  |  |
| Family study women, N (%) | 50 | 33 | 82 | 0.006 |
| Total dead, N (%) | 33 (66.0) | 13 (39.4) | 31 (37.8) | 0.006 |
| Age death (years) | 72.4 (14.1) | 79.4 (15.0) | 78.3 (11.7) | 0.071 |
| Age of Cardiovascular disease death (years) | 70.2 (13.5) | 80.3 (6.9) | 84.0 (7.4) | 0.008 |
| Age of Non-cardiovascular death (years) | 74.1 (14.7) | 80.3 (17.7) | 73.9 (12.0) | 0.496 |
| Cardiovascular disease, N (%) | 17 (51.5) | 4 (30.7) | 12 (37.5) | 0.367 |
| Non- cardiovascular death, N (%) | 16 (48.5) | 9 (69.2) | 19 (61.3) |  |
| Cancer death, N (%) | 6 (30) | 2 (10.0) | 12 (60.0) | 0.092 |
| Other death, N (%) | 10 (62.5) | 7 (77.7) | 7 (36.8) |  |

Data are summarized as mean (SD) or N (percentage). Test for raw differences using Chi^2^ test
